# Mechanisms Associated with Cognitive and Behavioral Impairment Induced by Arsenic Exposure

**DOI:** 10.3390/cells12212537

**Published:** 2023-10-28

**Authors:** Gustavo Ignacio Vázquez Cervantes, Dinora Fabiola González Esquivel, Daniela Ramírez Ortega, Tonali Blanco Ayala, Lucio Antonio Ramos Chávez, Humberto Emanuel López-López, Alelí Salazar, Itamar Flores, Benjamín Pineda, Saúl Gómez-Manzo, Verónica Pérez de la Cruz

**Affiliations:** 1 Neurobiochemistry and Behavior Laboratory, National Institute of Neurology and Neurosurgery “Manuel Velasco Suárez”, Mexico City 14269, Mexico; guigvace@gmail.com (G.I.V.C.); dinora.gonzalez@innn.edu.mx (D.F.G.E.); tblanco@innn.edu.mx (T.B.A.); emanuel25@ciencias.unam.mx (H.E.L.-L.); 2 Neuroimmunology Department, National Institute of Neurology and Neurosurgery “Manuel Velasco Suárez”, Mexico City 14269, Mexico; drmz_ortega@hotmail.com (D.R.O.); aleli.salazar@innn.edu.mx (A.S.); ifloresm1903@alumno.ipn.mx (I.F.); benjamin.pineda@innn.edu.mx (B.P.); 3 Departamento de Neuromorfología Funcional, Dirección de Investigaciones en Neurociencias, Instituto Nacional de Psiquiatría Ramón de la Fuente Muñiz, Mexico City 14370, Mexico; larch_chral@imp.edu.mx; 4 Laboratorio de Bioquímica Genética, Instituto Nacional de Pediatría, Secretaría de Salud, México City 04530, Mexico; saulmanzo@ciencias.unam.mx

**Keywords:** neurotoxicity, cognition, metalloids exposure, arsenic

## Abstract

Arsenic (As) is a metalloid naturally present in the environment, in food, water, soil, and air; however, its chronic exposure, even with low doses, represents a public health concern. For a long time, As was used as a pigment, pesticide, wood preservative, and for medical applications; its industrial use has recently decreased or has been discontinued due to its toxicity. Due to its versatile applications and distribution, there is a wide spectrum of human As exposure sources, mainly contaminated drinking water. The fact that As is present in drinking water implies chronic human exposure to this metalloid; it has become a worldwide health problem, since over 200 million people live where As levels exceed safe ranges. Many health problems have been associated with As chronic exposure including cancer, cardiovascular diseases, gastrointestinal disturbances, and brain dysfunctions. Because As can cross the blood–brain barrier (BBB), the brain represents a target organ where this metalloid can exert its long-term toxic effects. Many mechanisms of As neurotoxicity have been described: oxidative stress, inflammation, DNA damage, and mitochondrial dysfunction; all of them can converge, thus leading to impaired cellular functions, cell death, and in consequence, long-term detrimental effects. Here, we provide a current overview of As toxicity and integrated the global mechanisms involved in cognitive and behavioral impairment induced by As exposure show experimental strategies against its neurotoxicity.

## 1. Arsenic Generalities

Intoxication with arsenic (As) represents a public health problem since it is distributed throughout the Earth. As is a metalloid, considered the 53rd most abundant element on Earth’s crust [1], a constituent element of at least 245 minerals [2,3], in combination with lead, copper, and gold [2]. As (atomic number 33, molar mass of 74.92 g/mol) is considered a biophilic element because it can be found in organic materials [1]. According to the World Health Organization (WHO 2018), As toxicity is due to its wide distribution in groundwater, in polluted environments, and in food. As is the number one ranked element in the Substance Priority List of the Agency for Toxic Substances and Disease Registry due to the risk As poses as a potential hazard to human health [4]. To date, drinking As groundwater represents a major harmful risk in some countries, such as Bangladesh, India Myanmar, Argentina, Chile, China, Mexico, Vietnam, and Taiwan, and some states of the United States of America (Table 1) [5,6]. The US Environmental Protection Agency (USEPA) proposed a new standard for arsenic in drinking water of 0.005 mg/L, and in 2001, a standard of 0.01 mg/L was accepted [7,8,9]. Between 200 and 300 million people worldwide are exposed to As at higher concentrations, above the regulatory levels of 10 µg/L [10]. In fact, many countries have kept 50 µg/L as their national standard (Table 1).

Arsenic has been used since ancient times in pyrotechnics, medicine, agriculture, and the textile industry for pigmentation. Human exposure to As can occur from anthropogenic sources including mining waste, coal combustion, pigment production, and the use of As-based pesticides that contaminate both soil and drinking water sources [110]. In addition, the natural contamination of drinking water due to the As-metabolizing microbiome present in soil is also an important risk factor for human exposure [2]. Therefore, As intake into the human body is commonly due to the consumption of contaminated water or As-contaminated foods. Seafood, fish, and algae are sources of organic arsenic, increasing As levels in the bloodstream but then rapidly excreted unchanged in urine [111]. Long-term exposure to As can accumulate in tissues and increase the risk of cancer in different organs, including the lungs, bladder, and skin [6]. In fact, the International Agency for Research of Cancer (IARC) classified As as carcinogenic to humans; this metalloid has also been associated with cardiovascular disease [10], diabetes [112,113], neurological impairments [113], and developmental and reproductive alterations [114]. As can cross the placenta, causing impaired fetal development, miscarriages, and premature births [115]. These documented detrimental effects on human health due to long-term As exposure have led to more stringent regulations, as well as the encouragement to study of therapeutic alternatives to minimize its toxic effects. It is therefore important to know the mechanisms of As toxicity derived from experimental evidence with animal models, as well as data from the epidemiological studies and meta-analyses that have been conducted.

## 2. Speciation of Arsenic

**Figure 1:**
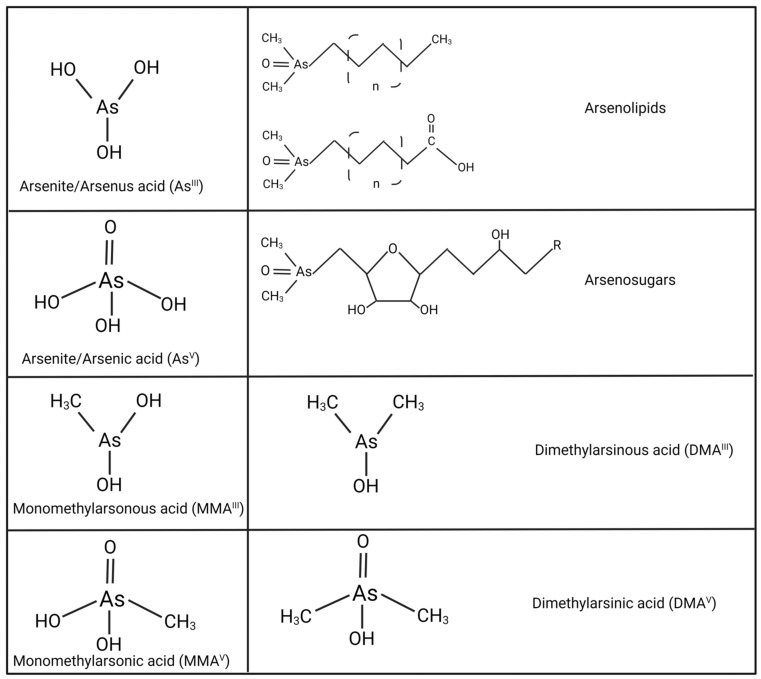
Arsenic species relevant to humans.

The different occurrence forms of As in fresh water depend on various factors, including redox potential, pH conditions, and As-stability. Arsenic is present in all possible oxidation states, +5, +3, 0, and −3, with arsenite (AsIII) and arsenate (AsV) being the most widely distributed. Among trivalent and pentavalent oxidation states, trivalent arsenic compounds are more toxic to mammals [116]. AsIII species are formed mainly under reducing conditions and at pH values lower than 9.2, while forming anions under higher pH values. Under oxidizing conditions and low pH, the AsV species predominate. As toxicity is also influenced by its metabolic transformation rate and its accumulation in tissues. Humans are exposed to both inorganic and organic forms of As, with the inorganic species being the most toxic [116,117]. The metabolic transformation of inorganic As species produces methylated As metabolites. Previously, methylation of iAs was interpreted only as a detoxification mechanism since it increases the rate of As clearance; however, more recently, methylation has been described as an activation mechanism required for at least part of the toxic effects of As [118]. Both arsenite and arsenate are coupled with methyl groups to form organic species such as monomethylarsonic (MMA) acid and dimethylarsinic acid (DMA). As can also be present in organic molecules when it is transformed into arseno-sugars or arseno-lipids [119,120] (Figure 1). At the present time, drinking water is considered the main source of As exposure, followed by dietary sources such as rice and seafood. The recognition of the association between exposure to As from environmental sources and adverse health effects in humans has led to its identification as a public health issue worldwide.

## 3. Arsenic Metabolism

After exposure to As, it is absorbed and distributed into the bloodstream to all the organs. Arsenate enters the cell via the phosphate carrier system, while arsenite uses aquaglyceroporins 3, 7, and 9 as transport system [121]. As mentioned earlier, the methylation of As occurs in all As species, and it plays an important role in the metabolism of iAs. Arsenic methylation by As methyltransferase (As3MT) is found in humans, rats, and mice. Experimental evidence using different mice strains showed that 96–99% of the total As urine samples present as dimethylarsinic acid (DMA^V^), indicating higher efficiency in metabolizing As compared to humans. Rats, on the other hand, sequester and retain arsenic in their erythrocytes, thus delaying its elimination, as shown by the 90-day long half-life of As in rats dosed with DMAs^V^ [122]. DMA^V^ is the most abundant arsenic metabolite in the urine of humans and most animals.

In the iAs metabolism, S-adenosyl methionine (SAM) is the methyl donor for iAs, producing monomethylarsonic acid (MMA^V^) and DMA^V^. This reaction is catalyzed by methyltransferases and requires glutathione as a co-factor [123]. After ingestion, AsV is reduced to AsIII by arsenate reductase, and then AsIII is methylated to MMA^V^ by SAM. Glutathione S-transferase-ω reduces MMA^V^ to MMA^III^, subsequently methylated to DMA^V^ by SAM, and finally reduced to DMA^III^ by a GSH-dependent enzyme. This process increases the cytotoxic effects of As, since DMA^III^ is 100–10,000 times more cytotoxic than DMA^V^ [124,125,126]. These As metabolites are excreted in the urine in a proportion of 10–15% MMA^V^, 60–80% DMA^V^, and 10–5% iAs, with an excretion range between 27 and 86 h [127,128].

In addition, Kala and coworkers found that As transport into bile is mediated by the MRP2/cMOAT transporter, and in a glutathione-dependent process, it is transformed to As-glutathione complexes, As-triglutathione (ATG) and monomethylarsonic diglutathione (MADG) [129]. Based on these findings, a new metabolic pathway for iAsIII was proposed, where the As-GSH complexes found in the bile are substrates for human arsenic methyltransferase Cyt19, then hydrolyzed and oxidized to MMA^V^ and DMA^V^ [130].

The methylation of iAs had been considered the major detoxification mechanism in most animals because organic arsenic cannot interact with some biomolecules due to its null ionization [131,132]. Other studies have found that some species of non-human primates lack hepatic arsenite methyltransferase activity [133]. Also, other species, such as the marmoset monkey, tamarins, guinea pigs, and chimpanzees, have methyltransferase deficiency, although they do express this enzyme’s genetic polymorphisms [134,135]. In mice, arsenite methyltransferase has shown tissue-specific activity rates with elevated enzymatic activity in the testis, liver, kidney, and lungs [136].

The protein binding of As has been studied in plasma samples of different species showing different values: ~20% in mice [137] and 10 to 50% in rabbits [138], while marmoset monkeys have the highest value, 70% [139]. In humans, this value was determined in the serum samples of ambulatory patients undergoing peritoneal dialysis; As-protein binding was only 5.5% of the total As found in serum. Only the iAs species bound slowly, ranging between 5.3% (AsIII) to 5% (AsV), and mainly to transferrin [140]. In vitro studies showed that iAsIII binds to sulfhydryl groups in proteins, while iAsV, at physiological pH, is present as an oxyanion with less affinity for protein binding. Those proteins with more cysteine residues have a higher affinity to iAsIII, as shown in a study demonstrating that arsenite preferentially binds to zinc finger peptides that contain three or four cysteine residues but not to peptides with only two cysteines [141].

The plasma pharmacokinetics of four As species in cynomolgus macaques were characterized after the intravenous and oral administration of arsenic trioxide solution (0.61 mg As/kg). The As species detected in plasma were AsIII, AsV, MMA^V^, and high levels of DMA^V^. The differential production of these As species involves As reduction and methylation in mammals, where gene polymorphisms of the enzymes involved in iAs biotransformation are suggested as the driving mechanism. This study then concluded that the absolute bioavailability of AsIII was 60.9%, confirming that this As-derived species is readily absorbed [142]. Previous reviews and metanalysis have addressed the main factors that can modify As metabolism [143,144,145]. Epidemiological studies in different populations showed large interindividual variations as well as the influence of doses, exposure time and diet, among others. For instance, Loffredo et al. studied methylation data from three different countries with high arsenic exposure areas: Mexico, China, and Chile, finding that DMA metabolite is present in higher concentrations in urine, with differences between males and females according to low–high exposure and genetic polymorphisms [146]. Furthermore, genetic comparisons of AS3MT (a key enzyme that produced methylated arsenicals transferring a methyl group to trivalent arsenicals) among three distinct ethnic Mexican groups with German and Asiatic populations found genetic differences in the distribution of 18 SNPs in this enzyme, particularly between the German and Mexican populations; in fact, the three ethnic Mexican groups studied, Mestizos, Nahuas, and Huicholes, also showed genetic heterogeneity [147].

Tian and Guo analyzed the data from the National Health and Nutrition and Examination Survey in the cycle 2015–2016 (NHANES), finding that Asians showed the highest blood and urine levels of eight biomarkers of four toxic metals, including arsenic in urine, among different race/ethnic groups in USA: Mexican American, non-Hispanic white, non-Hispanic black, other Hispanic, and non-Hispanic Asian. The main factor contributing to these differences was dietary habits (rice and seafood), in addition to socioeconomic and demographic influences [148].

Another source of chronic arsenic exposure in humans is through arsenic-contaminated air and food. In some regions of China, the use of arsenic-contaminated coal for cooking and crop drying has been reported. In some provinces, such as Guizhou and Shanxi, the As concentration in coal can be as high as 100–9600 mg/kg. The main serious health effects observed in people from the region which are caused by As are skin lesions (77.9%) that progresses to skin cancer. A high incidence of liver damage (40.97%), cirrhosis, and liver cancer are also reported. It has been estimated that 50–80% of arsenic exposure is due to contaminated food [149,150,151].

Recent studies have shown that methylated metabolites of As are genotoxic and cytotoxic in their trivalent oxidation state [152,153]. These trivalent metabolites showed a higher toxicity compared to their parental compounds in human liver cells [154], Chinese hamster ovary cells [153], and rat hepatocyte primary cultures [155]. This higher toxicity shown by trivalent methylated As species is related to their higher membrane permeability compared to pentavalent methylated species and iAs [153]. This toxic feature gains more significance if we consider that DMA is the major form of As transferred to the fetus and that As exposure during the early life stages has severe detrimental effects [156].

The fact that As can be metabolized and accumulated in the body suggests a difficult scenario in which several mechanisms co-exist and converge, leading to alterations in important cellular functions. A large number of both clinical and basic reports have demonstrated that long-term exposure to As leads to cognitive impairment; however, the mechanisms of As neurotoxicity have not been fully elucidated. The following sections describe murine models of As exposure and the mechanisms associated with cognitive impairment, in addition to the therapies that may attenuate the neurotoxicity of this metalloid evidenced by experimental research.

## 4. Cognitive and Behavioral Impairment Induced by As Exposure

Arsenic exposure is considered one of the environmental risk factors associated with several neurological diseases due to its permeability to the blood–brain barrier (BBB). In fact, it has been described that As can reach both the mature and fetal brain [156,157], accumulating and undergoing methylation in several brain areas and compromising brain function [158]. Humans exposed to As, both during early life and adulthood, showed cognitive impairment including learning and memory deficits [159,160,161]. Particularly, the detrimental effects of As exposure in the immature brain are not immediately apparent, but they may be reflected later in life with permanent effects such as intellectual dysfunction and motor and memory alterations, among others [160,162,163,164]. It is important to mention that most epidemiological studies have been carried out in children and adolescents exposed to arsenic during the early stages of life, and very few studies have been conducted in adults. Derived from a FRONTIER project study, it was evidenced that low-level arsenic exposure (current and long-term) was associated with lower scores in global cognition, a reduction in processing speed and immediate memory in rural-dwelling adults and elders, and symptoms that reflect or are common with the earliest manifestations of Alzheimer disease [165]. In addition, Chieng-Pang’s group found a strong correlation between Parkinson disease prevalence and arsenic levels in soil samples in three regions of Taiwan (Yunlin, Chiayi, and Tainan) [166]. Also, it was determined that As exposure increases the susceptibility for developing mood disorders. Derived from these findings, it has been proposed that exposure to As is a risk factor for late-life Alzheimer disease [161,165,167]. However, more epidemiological studies are necessary to confirm that the exposure to this metalloid is a risk factor for the development of Alzheimer or Parkinson diseases.

Due to the harmful health effects of chronic exposure to As in humans at different stages of life, various experimental models have been developed considering different exposure variables, such as concentrations, the time of exposure, and the life stage in which the exposure takes place (development, childhood, or adulthood). Table 2 summarizes the experimental models of exposure to As in rodents showing behavioral, cognitive, and motor alterations. Furthermore, redox, neurochemical, and structural changes are also summarized.

Evidence from various experimental models showed that alterations in signaling and function in the hippocampus are associated with As exposure [158].

Gestational or perinatal models of As exposure represent particularly vulnerable stages during brain development, as the BBB is immature and As can easily pass through it, thus interfering with relevant processes that are determinant for cognition such as neuronal plasticity and the establishment of the neuronal network. Experimental models of perinatal exposure to As in pregnant females and during the pup lactation period did not find changes in eye-opening, pinna detachment, incisor eruption, or the growth of fur [207,208]. However, As exposure increased the time to complete reflexive responses (setpoints of good neurological development such as the surface righting reflex, negative geotaxis reflex, and cliff avoidance reflex) in the pups, demonstrating alterations in both motor function and sensory inputs [183,207,209]. In other perinatal models, As exposure induced spatial memory and social behavior impairments, as well as increased anxiety-like behavior [169,177,178,208]. Cognitive and behavioral deficits induced by As exposure during critical periods of neurodevelopment are long-lasting. Exposure to As in adulthood can also lead to alterations in locomotor activity, learning disabilities, and memory deficits (Table 2). Adult female mice intoxicated with As_2_O_3_ for 45 days showed a reduction in explorative activity, increased anxiety levels, and learning and memory impairments [189]. In this line, male rats exposed to As during gestation until PND21 showed a severe learning memory impairment compared to females rats [210]. Recently, the following was observed in adult male rats exposed to iAs: altered social interaction behavior (sociability and social memory), disabilities in motor coordination, impairments in the performance of the novel object recognition test as well as in spatial learning, and increased anxiety-like behavior [189,206]. Controversially, rats exposed to As during their early life showed hyperactivity (distance traveled, moving time, and resting time), which was also associated with an increase in dopamine D2 receptor expression in the striatum [211]. These discrepancies in cognitive and motor parameters found after exposure to As are due to the varying doses, duration, and route of arsenic exposure, as well as the experimental model used (gestational, lactation, or adulthood). Cognitive impairment and alterations in behavior and social interaction are part of the detrimental effects caused by As exposure, which are associated with brain morphological changes, mainly in the hippocampus, a brain region important for regulating cellular processes that drive cognition. Some of these morphological changes are described below.

## 5. Morphological Alterations Induced by As Exposure

In this section, we summarize the brain histopathological alterations induced by As exposure that have been shown in experimental models. Several structural alterations described after As exposure compromise long-term brain function, especially if the exposure occurs during early life. For instance, it has been shown that brain weight decreases around 13–15% after 45 days of As exposure [189]; this change could be associated with the loss of brain cells, thus leading to cognitive performance decline and behavioral and motor impairments, as described previously [212]. It is worth noticing that morphological, biochemical, and molecular alterations observed after the exposure to As are different between brain regions, which could be in part related by the fact that the distribution of arsenic species in the brain is differential and depends on the doses of As and the gender. A study described that the accumulation of As in the brain is as follows, using doses of 5 mg/kg of NaAsO_2_: hippocampus > cerebral cortex > cerebellum. When the doses are higher (20 mg/kg), the distribution is cerebral cortex > hippocampus > cerebellum [213]. Notably, the concentration of iAs and MMA were extremely higher in the hippocampus compared to cerebral cortex and cerebellum, which correlate with the learning and memory alterations found in several studies. Also, subchronic exposure to As decreases the levels of essential trace elements in the CNS (Fe, Se, Zn, and Cr) in the cerebrum and cerebellum of mice. Noteworthy, this effect was shown to be also influenced by gender, particularly the effect of As on the concentration of Se in the cerebrum and Cu and Se in the cerebellum. However, more experiments are needed to confirm and explain the mechanisms leading to these differences [214,215].

Regarding the fact that the developing brain is a target for As toxicity with greater detrimental effects, there is more extensive evidence about morphological changes during this period of time; however, the detrimental effects of As that occur in later stages of life are also significant [208,209,216,217].

Luo and coworkers showed morphological alterations in hippocampus cells when weaning rats that were chronically exposed to As. These morphological changes included cells with condensed cytoplasm and nucleus, swollen mitochondria, disorganized cellular arrangement, vacuolar degeneration, and edema. In addition, at the synapse of hippocampal neurons, a reduced number of synaptic vesicles were observed [185]. In addition, neonatal rat brains previously exposed to As during gestation showed the degeneration of the cytoplasm as well as the loss of cell–cell junctions [218]. Furthermore, rat pups exposed to sub-acute iAs intoxication lost the Purkinje cell monolayer, whereas the remaining cells showed delayed maturation and increased nuclear area [219].

In adult mice exposed to As chronically, morphological changes in the hippocampus such as the loss of the hippocampal packing arrangement of pyramidal neurons, an indistinct nucleolus, and neurons with pycnotic and fragmented nuclei were observed [188]. In the same study, a reduction in neuronal density and cell layer thickness was found after As_2_O_3_ exposure [188]. In vitro evidence has also shown that arsenite reduced neurite production, outgrowth, and complexity in newly differentiating PC12 cells [220]. Furthermore, in a chronic in vivo model with rats exposed to As from gestation to adulthood, alterations in cortical myelination and mitochondrial mass were found, while a decrease in white matter volume and an increase in radial diffusivity indicated that As exposure altered white matter tracts [221]. Nuclear pyknosis, cellular shrinkage, and cytoplasmic disintegration were observed after the infusion of As (5 nmol) into the substantia nigra of rats’ brains [222].

In addition to neuronal damage, As exposure induces the loss of astrocytic morphology, reducing the number of astrocytic projections, decreasing GFAP expression, and increasing MMP9. Additionally, perinatal As exposure induces astrocytic apoptosis through increased PARP activity during the developmental stages, which is associated with blood–brain barrier disruption and behavioral deficits [223,224,225].

In the next section, the neurotoxic mechanisms induced by exposure to this metalloid will be described, as well as approaches in experimental models that could be taken into consideration in the future.

## 6. Mechanisms Involved in the Neurotoxicity Induced by As Exposure

As mentioned above, the toxic effects of As species during the early life stages becomes an important risk factor for the development of neurological and cognitive deficits. In this context, several studies report cognitive deficits in children exposed to As sources [226,227,228]. For this reason, clarifying and integrating the mechanisms of As neurotoxicity and cognitive dysfunction during the lifespan could contribute to finding new therapeutic targets that improve the quality of life of people exposed to this metalloid. Herein, we provide information about the mechanisms associated with As exposure in different experimental models.

### 6.1. Redox and Mitochondrial Alterations Related to Arsenic Exposure

The brain is particularly vulnerable to redox environment alterations, due to its high rate of oxygen consumption and polyunsaturated fatty acid content [229]. In this sense, one of the most studied mechanisms of As neurotoxicity is oxidative stress. Oxidative stress is related to mitochondrial dysfunction, protein and DNA alterations, neurochemical changes, and neuronal death, all of which may converge and contribute to cognitive dysfunction.

Moreover, the high energy requirements of the brain depend on mitochondrial ATP production. During this mitochondrial energy production process, free radicals, reactive oxygen species (ROS), and reactive nitrogen species (RNS) are produced simultaneously. In early developmental stages, these ROS are considered essential for brain development and function [230]; however, when their production is excessive and/or the antioxidant defense is diminished, an oxidative stress environment is favored. Therefore, protein oxidation, lipid peroxidation, and DNA fragmentation are triggered, ultimately leading to cellular damage. In this line, As neurotoxicity has also been linked to redox balance disruption in the brain through generating free radicals, decreasing the antioxidant capacity, inducing mitochondrial dysfunction, and promoting cell damage.

In vitro evidence, using bovine milk xanthine oxidase, showed that AsIII can be converted to AsV via oxidation, generating two electrons that favor the production of hydrogen peroxide [231]. Although further in vivo experiments are needed, the authors proposed that H_2_O_2_ production by xanthine oxidase may be an important route for decreasing the toxicity of trivalent arsenic species through oxidizing them to their less toxic pentavalent analogs [232].

Also, as we previously mentioned, inorganic arsenic is metabolized to their methylated forms as a detoxification mechanism; when this process occurs, it has been demonstrated that dimethyl arsine, a further metabolite of DMA, can react with molecular oxygen producing dimethylarsinic radicals and superoxide anions [233]. Of note, this reaction can be inhibited by superoxide dismutase (SOD) or catalase enzymatic activity. The dimethylarsinic radical reacts with a second oxygen molecule to produce DMA peroxyl radical ((CH_3_)_2_AsOO•) [234,235].

A recent study showed mechanistic differences between As species (iAsIII, MMAIII, and DMAIII). Methylated trivalent arsenicals, MMA III, and DMAIII at low concentrations (1 µM) are the earliest to produce intracellular ROS, while higher concentrations of iAsIII are required to increase ROS at a later time compared to their methylated derivates. Furthermore, the intracellular location of ROS formation induced by these methylated species was differential: MMAIII produces ROS in mitochondria, whereas ROS production by DMAIII takes place preferentially in the ribosome, endoplasmic reticulum, and Golgi apparatus [236]. In addition, arsenic species can induce the release of iron from ferritin (AsV > AsIII > MMAV > MMAIII > DMAV > DMAIII), a process that promotes the iron-dependent formation of ROS [237].

The Induction of a redox unbalance by As also involves glutathione (GSH), which is a tripeptide (glutamic acid, cysteine, and glycine) considered the major antioxidant in the brain. The antioxidant properties of GSH are due to the presence of the sulfhydryl group in the cysteine moiety [238]. To eliminate ROS and/or RNS, two molecules of reduced glutathione (GSH) are oxidized, generating one molecule of oxidized glutathione (GSSG), which is later reduced by glutathione reductase (GR) to regenerate GSH [239]. Arsenic depletes GSH content through several mechanisms: (1) the GSH-dependent reduction of arsenate to arsenite, where GSH serves as the electron donor to the reaction producing GSSG [240]; (2) arsenite and its methylated metabolites inhibit the GR, generating an imbalance in the GSH/GSSG ratio [241,242]; (3) As induces excessive ROS that GSH needs to detoxify, also disrupting the balance GSH/GSSG; and (4) As is bound to GSH to form an As-GSH complex to expel As from cells [243]. In vitro studies in primary astrocytes demonstrated that both AsIII and AsV increased GSSG levels, while GSH and the mitochondrial membrane potential were decreased [244,245]. This negative cycle can be favored by the binding of trivalent arsenicals to thiol groups on key antioxidant enzymes inhibiting their function, such as GR, glutathione peroxidase, thioredoxin peroxidase, and thioredoxin reductase [246,247].

At this point, it is important to consider that the mitochondrion is one of the major ROS generators and another target for arsenic toxicity. In fact, inorganic arsenicals induce the inhibition of mitochondrial respiration. In this line, it was shown that MMAIII exposure induces a strong inhibition of mitochondrial complex II and IV, while DMAIII inhibited complex II [236], which in turn enhances ROS production and compromises ATP production. Notably, energy metabolism is affected by As exposure through inhibiting pyruvate dehydrogenase (PDH), a critical enzyme that catalyzes the decarboxylation of pyruvate to acetyl-CoA, the link between glycolysis and the citric acid cycle. PDH inhibition by As leads to the inhibition of the citric acid cycle, in which reducing equivalents needed for ATP production are generated [248].

In addition, ROS production induced by As depends on the time of exposure and concentration, since low concentrations of As generate an extremely rapid ROS production in the mitochondria, while higher concentrations induce a slower effect that involves calcium mobilization from the endoplasmic reticulum (ER) to the mitochondria, thus altering calcium homeostasis [249,250,251]. Calcium mobilization and accumulation in these organelles after As exposure implies that many processes are altered favoring the toxicity of this metalloid. Among these processes are the induction of the mitochondrial permeability transition, a loss in mitochondrial membrane potential, an increase in mitochondrial ROS, and an elevation in intracellular free-calcium—mitochondrial events that together result in pro-apoptotic responses (cytochrome C release, the activation of caspase 3 and 9, and increases in Bax/Bcl-2) [252,253]. In vivo evidence has shown that mitochondrial membrane potential as well as mitochondrial activity complexes are decreased in the striatum when rats are exposed to As (from 22 to 59 postnatal day) [211]. These mitochondrial alterations occur in parallel to other redox alterations, such as increased ROS/NOS, protein carbonylation, and lipoperoxidation, as well as a decrease in GSH content and reduced antioxidant enzyme activity (CAT, GPx, and SOD) [177,218,254].

Mitochondrial biogenesis is also impaired by As exposure, where the peroxisome proliferator-activated receptor-γ coactivator (PGC)-1α is the main regulator [255]. Mitochondrial biogenesis starts with the activation of PCG-1α through its deacetylation or phosphorylation; then, nuclear respiratory factors 1 and 2 (NRF-1 and NRF-2) are stimulated, leading to an increase in mitochondrial transcription factor A (Tfam), which is the final inducer of mitochondrial DNA transcription and replication. In the rat brain, chronic exposure to sodium arsenite decreases the expression and activity of mitochondrial respiratory complexes and the expression and protein levels of PGC-1α, NRF-1, NRF-2, and Tfam, while increasing Bax, caspase 3, and cytochrome c proteins, thus suggesting the induction of cellular death through apoptosis [256]. An integrative analysis of the metabolome and proteome of the cortex of As-exposed rats yields potential biomarkers related to cognitive impairment caused by this metalloid; these included the overexpression of apolipoprotein E, decrease in mitochondrial complex IV, alteration of SOD1, dysregulation of GSH metabolism, and increased of heterogeneous nuclear ribonucleoprotein L (hnRNP L, an RNA processing factor, and an RNA-binding protein) expression [257].

This vicious circle between mitochondrial alterations and oxidative stress leads ultimately to cell death. However, before neuronal death occurs, if the cellular energy provided by the mitochondria is altered, proper neurotransmission will also be compromised, which together will result in an alteration of the correct functioning of the central nervous system (CNS).

### 6.2. Neurochemical Dysfunction Induced by Arsenic Exposure

In addition to the oxidative and mitochondrial alterations found after As exposure, alterations of neurotransmitter equilibrium and synaptic plasticity in the brain have also been described. These alterations include a reduction in: (1) the expression of dopaminergic receptors, reduced dopamine levels, and its metabolites (homovanillinic acid and 3,4-dihydroxyphenylacetic acid); (2) acetylcholine production and acetylcholinesterase activity; (3) the expression of the *N*-methyl-d-aspartate receptor (NMDAr) subunits NR1, NR2A, and NR2B; 4) the expression of the α-amino-3-hydroxy-5-methyl-4-isoxazolpropionic acid receptor (AMPAr); and (5) cysteine and glutamate transporters [177,178,183,222,258,259]. The fact that As exposure induces alterations in NMDAr and AMPAr expression would explain one of the mechanisms through which it unables synaptic plasticity and cognition. These alterations in NMDAr subunits have been elucidated in mice using an intoxication developmental model where the hippocampal NR1, NR2A, and NR2B subunit expression decreased in an As dose-dependent manner (25, 50, and 100 mg NaAsO_2_/L). Also, ionotropic glutamate receptor subunits GluR1, GluR2, and GluR3 expression decreased gradually after As exposure. These changes in the NMDAr and AMPAr subunits were associated with a decrease in calcium/calmodulin-dependent protein kinase II (CAMKII) and phosphorylated-CAMKII in the hippocampus. This evidence suggests that As leads to NMDAr and AMPAr dysfunction, which in consequence impairs long-term potentiation and cognitive impairment [179].

Notably, in vitro studies have demonstrated the detrimental role of As in different cell types of the CNS and described different effector mechanisms. It has been reported that human brain cell cultures, including neurons, primary astrocytes, microglia, and brain microvascular endothelial cells, are more sensitive to methylated As species such as methylarsenite [223,260]. In addition, it has been reported that As can reduce neurite growth and differentiation through decreasing the expression of AMPAr on primary cortical neurons [220,261]. The harmful effects of As exposure in in vitro studies include the reduction in the expression of matrix metalloproteinases (MMP) 2 and 9 and the microtubule-associated protein doublecortin [262]. Also, primary cortical neurons exposed to 5 µM of As have been shown to exhibit decreased levels of α-synuclein, a protein involved in the regulation of neurotransmission, together with enhanced auto-phagocytic activity that eventually led to neural death [263]. Apart from the harmful roles of As on neurons, As-mediated toxicity in other brain cell types could potentiate the dysfunction observed in the CNS. In this line, astrocytes have shown to be more sensitive to AsIII than to AsV [244]. As-mediated toxicity was associated with a decrease in glial fibrillar acidic protein (GFAP) expression and the attenuation of the heparin-binding epidermal growth factor (EGF)-like growth factor (HB-EGF)/EGFR signaling axis, which in turn mediated the overexpression of the MMP-9 [224]. As also increased the expression and cleavage of the poly-ADP-ribose polymerase (PARP), promoting the activation of pro-apoptotic signals [25]. In addition to apoptosis induction, As also decreased the expression of glutamine synthetase, glutamate-aspartate transporter (GLAST), and glutamate transporter (GLT)-1, disrupting the metabolism of glutamate in astrocytes that play a critical role in the regulation of glutamatergic neurotransmission [245].

On the other hand, even though As at nanomolar concentrations does not affect the viability of microglial cells, the innate immune component of the brain, it can activate the c-Jun N-terminal kinase (JNK)/signal transducer and activator of transcription (STAT)3/nuclear factor-κB (NF-κB) in these cells, increasing the production of pro-inflammatory cytokines such as interleukin (IL)-1β, IL-6, and tumor necrosis factor α (TNF-α), reducing phagocytic activity and increasing the expression of Iba-1, a microglial activation marker [264,265,266,267]. The As-mediated effects on astrocytes and microglia have repercussions on neuronal viability, it has been reported that neurons incubated with conditioned mediums of As-treated astrocytes had fewer presynaptic terminals, as well as a decreased expression of the NMDAR subunits NR1, NR2A, and NR2B and reduced levels of adenylyl cyclase and Calcium/calmodulin-dependent protein kinase III (CaMKIII) [268]. Moreover, As exposure induces the production of proinflammatory cytokines secreted by microglia in conditioned media, which can induce endoplasmic reticulum stress, which in turn leads to apoptosis in neurons in vitro [265,266]. It is worth mentioning that As exposure has been related to Alzheimer’s disease, since DMA^V^ modifies APP processing in vitro. It has been proposed that chronic As exposure during development favors the amyloidogenic pathway through an increase in APP expression and soluble Aβ(1–42) levels [269].

Furthermore, cell adhesion molecules involved in cell migration and neuronal growth are altered after As exposure. In this context, rat offspring exposed to As during gestation and lactation showed decreased expression of neural cell adhesion molecules (NCAM) and polysialyl transferases involved in the regulation of synaptic connections during neuronal development, which could explain the cognitive alterations they exhibited [209]. Consisting with and complementary to this approach, the effect of perinatal As exposure on memory deficits has been associated with decreased D-serine metabolism. This amino acid acts as a co-agonist of NMDA receptors for synapse establishment and long-term potentiation during neurodevelopment. D-serine decrement by As was accompanied by the reduced expression of NMDAr subunits [270].

Another emerging alteration induced by As exposure involves neuroendocrine signaling. Studies had associated social and cognitive dysfunctions after As exposure with reduced levels of the corticotrophin-releasing factor receptor (CRFR1), glucocorticoid and mineralocorticoid receptors, indicating a disruption of the hypothalamic–pituitary–adrenal stress axis [173,174].

Notably, another study focused on neurochemical alterations induced by As exposure during early life showed that the amines noradrenaline, dopamine (DA), and serotonin (5-HT) decreased in the cerebellum and hypothalamus. Of note, these alterations persisted for a long period of time after cessation of As intoxication. In contrast, an increase in 5-HT was observed in the striatum and hippocampus, which was restored after the discontinuation of exposure. Meanwhile, in the motor cortex and hippocampus, DA levels were altered even when the exposure stopped. Additionally, when glutamic acid decarboxylase (GAD) activity and GABA levels were measured, a notable reduction in these markers was observed in the cerebellum, hypothalamus, and brainstem of rats exposed to As. Surprisingly, glutamate had no changes compared with the control group. Conversely, using a different approach, when As exposure occurred during adulthood through diet, glutamate increased in the motor cortex, striatum, nucleus accumbens, and hippocampus; GABA levels were reduced in the striatum, cerebellum, and brainstem; and GAD activity was reduced in the hippocampus, hypothalamus, and cerebellum [271]. According with these findings, Hu and coworkers recently reported that sub-chronic As exposure induces a reduction in the expression of both GAD and GABA_B2_ receptors in the prefrontal cortex correlated with anxiety-like behaviors [272].

**Figure 2:**
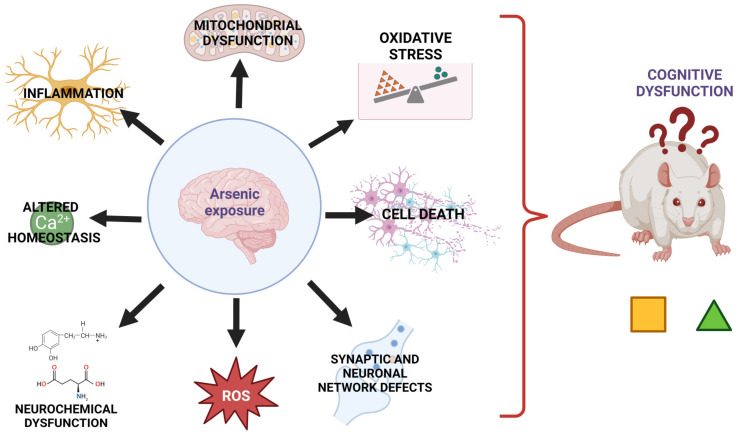
Arsenic mechanisms involved in the induction of neurotoxicity. Acute or chronic As exposure induces neurotoxicity via different mechanisms that may interact with each other. As-induced mitochondrial dysfunction leads to excessive production of ROS that may leak from mitochondria favoring an oxidative stress environment, which in turn may alter Ca^2+^ homeostasis and induce neurochemical dysfunction. If As exposure occurs early in life or during neurodevelopment, synaptic and neural network impairment is triggered. As can also alter microglial functions and overactivate NF-κB-mediated signaling and thereby increase the production of proinflammatory cytokines (IL-6, IL-1β, TNF-α). Taken together, these As-induced neurotoxic mechanisms may ultimately result in neuronal death and cognitive and behavioral alterations.

Neurochemical, protein, redox, and molecular changes induced by arsenic exposure in the early stages of neurodevelopment or during adulthood have a detrimental effect even after the discontinuation of As exposure (Figure 2). However, some of these deleterious effects induced by As toxicity can be mitigated using several strategies described in the next section.

## 7. Strategies against As Toxicity

### 7.1. Arsenic Poisoning Treatment

Prolonged exposure to As through ingestion, inhalation, or dermal contact can induce a wide spectrum of detrimental effects on human health including dermatitis, cardiovascular and blood diseases, neurotoxicity, nephrotoxicity, and several types of cancer. After being exposed to As, the first line of therapy is symptomatic and supportive treatment including chelation and antioxidant therapies.

The common treatment for acute and severe cases of As poisoning is chelation therapy. Chelation is a process in which ions or molecules of a ligand donate a pair of electrons to form a covalent bond to central metal atoms in a cyclic or ring structure. Chelators or chelating agents bind to the heavy metal molecules in the bloodstream and, later, they are filtered out through the kidneys and excreted in the urine. An ideal chelating agent should have chemically inert and non-toxic complexes with metal atoms or ions, should be easily excreted from the body without interacting with any biomolecules or vital organs, should penetrate the cell membrane to remove intracellular toxic metals and, to counteract neurotoxicity, should have high lipophilicity and low molecular size to be able to cross the BBB [273].

One of the most used chelating agents is DMPS (dimercaptopropane sulfonate) or DMSA (dimercaptosuccinic acid) derived from dimercaprol or BAL (British anti-Lewite, dimercaptopropanol), which are water-soluble and less toxic. DMPS contains one sulfonic group and two thiol groups and, due to its water-soluble nature, does not readily cross the BBB, so it cannot remove heavy metals or metalloids from brain tissue. However, it effectively removes heavy metals and metalloids from the kidneys and has mild side effects compared to BAL. DMSA is another BAL analog with fewer side effects; this compound contains two carboxylic groups and two thiol groups, with the latter responsible for the metal-ligand reaction. Recently, several DMSA analogs have been developed—MiADMSA (monoisoamyl DMSA), MchDMSA (monocyclohexyl DMSA), and MmDMSA (monomethyl DMSA)—which show higher arsenic chelation efficiency and better excretory efficiency than DMS. Moreover, due to their lipophilic nature, they can penetrate cells and pass through the BBB and markedly decrease the As content in most of the organs as soon as 1.5 h after administration. Penicillamine, another chelating agent, is also used as a second or third agent for the treatment of As toxicity [273].

However, although these chelating agents have the ability to expel heavy metals and metalloids such as As from the body, they cannot restore cellular antioxidant defenses and redox balance in tissues in which As has induced oxidative stress. A therapeutic strategy that restores the antioxidant capacity of these cells may be essential to provide effective long-term treatment of As poisoning [274]. A recommended alternative to counteract this oxidative damage is the supplementation of antioxidant compounds such as N-acetylcysteine (NAC), taurine, melatonin, α-lipoic acid, and vitamins A and E. These compounds function as direct ROS scavengers, can stimulate GSH synthesis, restore the prooxidant/antioxidant balance, and act as As chelators (e.g., NAC). Most of these antioxidants can be administered directly as safe pharmacological drugs or can also be derived from a natural source through our diet [274].

### 7.2. Nutritional Interventions and Natural Compounds

Descriptive reviews about nutritional interventions and natural compounds used to ameliorate the deleterious effects of arsenic toxicity have been published recently [275,276]. Here, we summarized some of this evidence and the shared mechanisms of these compounds. Medicinal plants have been used worldwide as treatments for a variety of diseases and as raw materials to produce phytopharmaceuticals. Compared to the side effects reported with other treatments for arsenicosis such as chelation therapy, natural products have shown effectiveness in treating some of the effects of As toxicity without showing adverse side effects. Natural products exhibit a common mechanism of action through counteracting the pro-oxidant stress induced by As, since most of these natural dietary compounds show antioxidant properties. These antioxidant properties are associated with its high content of hydrophilic phenolic compounds, especially flavonoids.

**Figure 3:**
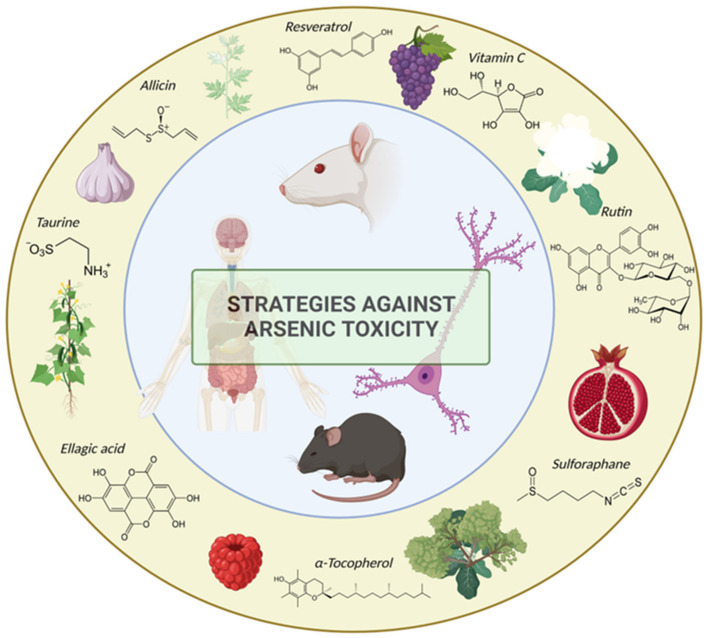
Strategies against arsenic toxicity: natural compounds. Natural products have a common mechanism of action, counteracting As-induced prooxidant stress. Most of these natural dietary compounds show antioxidant properties due to their high content of hydrophilic phenolic compounds, as we can see in the representative chemical structures of some of these molecules: Resveratrol, vitamin C, rutin, sulforaphane, α-tocopherol, ellagic acid, and taurine, as well as allicin. These compounds can be found in medicinal plants or in vegetables such as cauliflower, broccoli, and garlic, or in fruits such as grapes and raspberries.

A literature review by Bhattachary” addresses the preclinical and clinical studies on medicinal plants and natural products showing evidence of significant protection against As toxicity [277]. A total of 34 medicinal plants exhibited experimental evidence of ameliorating As toxicity in sub-chronic models; most of the studied plants belong to the traditional system of Indian medicine and similar systems worldwide [277]. Some examples of medicinal plants shown to significantly reduce As toxicity are *Allium sativum* (garlic), *Ginkgo biloba* (ginkgo), and *Curcuma longa* (turmeric). Some of these medicinal plants are rich in vitamin C, vitamin B6 manganese, and organosulfur compounds (e.g., allicin for garlic) [278]. The mechanisms of protection against As toxicity include the ability of these compounds to combine with heavy metals and metalloids in the body, favoring their excretion in the feces, thus reducing their circulating levels and preventing their accumulation in soft tissues including the brain; sulfur compounds such as diallyl sulfide, present in garlic, have been identified as responsible for this property (Figure 3). Additionally, these compounds are radical scavengers, so they can reduce oxidative stress, suppress lipid peroxidation, and elevate intracellular glutathione levels. Histopathologic damage is also reduced by these compounds through the inhibition of the serum alkaline phosphatase enzyme. Chelating properties and the ability to enhance the activity of catalase, glutathione antioxidant system, and superoxide dismutase enzymes also contribute to their protective profile against As toxicity.

Several isolated natural compounds present in medicinal plants have shown ameliorative effects against As toxicity in animal models and in vitro studies, among which the following have the greatest amount of experimental evidence in the context of neuroptotection (an extensive list can be found in Bjørklund et al., 2022 [275], and Najafi et al., 2023 [279]): Alpha and dihydrolipoic acid (α-LA and DHLA), curcumin, D-pinitol, hydroxytyrosol, ellagic acid, glycyrrhetinic acid, luteolin, lycopene, quercitin, taurine, thymoquinone, epigallocatechin-3-gallate, sulforophane, and α-tocopherol. The ameliorative efficacy of these compounds includes their ability to reduce inflammation and As levels in the brain tissue; modulate cell death mediated via apoptosis or autophagy; reduce lactate dehydrogenase levels and caspase 3 activity; induce SOD, CAT, γ-glutamylcysteine synthetase (γ-GCS), and GSH-peroxidase activity; boost GSH intracellular levels; reduce intracellular ROS and MDA levels; reduce mitochondrial dysfunction and restore ATP synthesis; prevent As-induced DNA fragmentation; block NF-ƘB pro-inflammatory signaling pathways; and activate the Nrf2-signaling pathway [280,281,282,283,284,285,286,287,288]. Other compounds, such as ellagic acid, resveratrol, rutin, and taurine, deserve a separate mention, since, in addition to the neuroprotective effects mentioned above, they also ameliorate As-induced cognitive and behavioral impairments in in vivo rodent models [193,204,206,289]. Recently, Dictyophora polysaccharide, a bioactive compound from medicinal and edible fungus, has been used as a tool against As neurotoxicity due its anti-inflammatory and immunomodulatory activities [290,291].

Moreover, an appropriate diet and nutrition can prevent As-related disorders mainly because nutrition deficiency can delay the removal of As from the body. An adequate diet that includes vegetables from the genera Brassica, such as cauliflower, broccoli, and turnip, rich in organosulfur compounds, is useful to eliminate As from the liver (Figure 3). Essential micronutrients such as selenium, calcium, and magnesium have also been studied as potential mitigators of As toxicity. Finally, among these mineral compounds, selenium has shown a greater effect on reducing oxidative stress; this effect has been mainly attributed to its role as a component of glutathione peroxidase, an important antioxidant enzyme that reduces the production of ROS [292].

However, in addition to these potentially therapeutic interventions, it is important to say that if we consider that, according to the WHO, an estimated 140 million people in at least 70 countries have been drinking water with arsenic levels above guideline values (10 µg/L), the prevention of further exposure to arsenic represents the best option to avoid the long-term effects induced by this metalloid: test water to discriminate between high-arsenic sources from low-arsenic sources, install arsenic removal systems, reduce occupational exposure from industrial processes, and monitor high-risk populations for early signs of arsenic poisoning (World Health Organization Home Page. Available online: https://www.who.int/news-room/fact-sheets/detail/arsenic (accessed on 23 October 2023)).

## 8. Concluding Remarks and Perspectives

In this review, we described the sources of As, its metabolism and distribution, and, more importantly, its health implications and the mechanisms associated with its neurotoxicity and long-term consequences. A few key points are noteworthy: (a) Arsenic is a widely distributed metalloid commonly found in polluted water, soil, air, and food. (b) Arsenic detoxification occurs mainly through methylation and the formation of GSH-adducts and protein-thiols. (c) Inorganic and methylated arsenic species can accumulate in different tissues including the brain, in which the accumulation is region-specific. (d) Perinatal exposure to arsenic leads to long-term behavioral, cognitive, and motor deficits. (e) Limited epidemiological studies are focused on chronic exposure to arsenic during adulthood and its relationship with neurodegenerative diseases such as Alzheimer and Parkinson diseases. (f) Extensive evidence from experimental models has characterized the common mechanisms through which As induces neurological damage. (g) The neurotoxic mechanisms of arsenic include exacerbated oxidative stress, mitochondrial dysfunction, and impaired synaptic activity, which drive cell damage and, ultimately, cell death. (h) Chelating agents are effective molecules for arsenic detoxification that can be combined with natural products (mainly organosulfur compounds) that may restore the cellular antioxidant machinery.

Finally, the available studies indicate that arsenic exposure results in cognitive and behavioral impairments; however, these long-lasting effects are the consequence of several mechanisms triggered by this metalloid, such as oxidative stress, mitochondrial dysfunction, and neurochemical and molecular alterations, which converge and affect important processes, such as the establishment of neural networks, proliferation, migration, and plasticity, leading to cell death and cognitive dysfunction. The challenge of future research is to be able to modulate the targets of toxicity of this metalloid to attenuate its toxic effect and ultimately improve the health of high-risk populations exposed to this metalloid.

## Figures and Tables

**Table 1:** Consequences associated with As exposure described in different countries.

Reported Alterations Associated with As Exposure	Country	Regulatory Levels (mg/mL)	As Levels Exposure	People Exposed	References
Fetal development effects (low birth weight and short birth height) Spontaneous abortions Higher risk of birth defects (nervous system deformity, limbs deformity and congenital heart disease) Neurodevelopment alterations Reduced intellectual function Gestational diabetes Nerve velocity alterations Neuropathy Memory and cognitive impairment Acute psychosis with hallucinations Cardiovascular abnormalities Keratosis and hyperpigmentation Gastrointestinal alterations Type 2 diabetes and prediabetes Cancer (bladder, lung, kidney, skin, liver, stomach and rectal) Infertility Sleep disturbances Respiratory diseases Oxidative stress Dysglycemia Alterations in DNA repair mechanisms and DNA damage Changes in methylation Anemia Blackfoot disease Renal dysfunction Lung function impairment Hyperglycemia Alterations in inflammatory markers Endocrine disruption Liver damage Changes on the redox balance Leukopenia and lymphocytosis Dyspepsia Phospholipids, phenylalanine, and tryptophan metabolism alterations Alterations in inflammatory markers	Canada	0.01	0.0005–3.78	1,700,000	[11,12,13,14,15,16,17,18,19,20,21]
	United States of America	0.01	0.01–12	44,100,000	[11,21,22,23,24,25,26,27,28,29,30,31,32,33]
	Mexico	0.25	0.008–1.1	2,000,000	[11,34,35,36,37,38,39,40,41,42,43,44,45,46,47,48,49,50,51,52,53]
	Chile	0.05	0.05–2	2,100,000	[11,21,54,55,56,57,58,59,60]
	Argentina	0.05	0.004–14.969	3,500,000	[61,62,63,64]
	China	0.01	0.01–126	33,000,000	[65,66,67,68,69,70,71,72,73,74,75]
	Vietnam	0.05	0.001–3.05	10,000,000	[11,76,77,78,79,80,81]
	India	0.05	0.0005–3.2	45,000,000 to 100,000,000	[76,82,83,84,85,86,87,88]
	Bangladesh	0.05	>0.05–4.6	85,000,000	[21,89,90,91,92,93,94,95,96,97,98,99,100,101,102,103,104,105]
	Spain	0.05	0.001–0.29	50,000	[21,76,106,107,108,109]

**Table 2:** Experimental model of arsenic exposure.

Exposure Model	Doses	Cognitive/Behavioral Alterations	Redox, Neurochemical, and Structural Alterations	References
Gestational Exposure 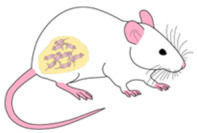	Pregnant C3H mice exposed to NaAsO_2_ (85 ppm) from GD 8 to GD 18.	Impaired adaptation to repetitive reversal tasks in adulthood (60 postnatal weeks) Poor sociability and poor social novelty preference at 74 postnatal weeks on F1, as well as on F2.	Increase in the number of pyramidal neurons in layers V and VI of the prelimbic cortex.	[168,169,170]
			Decreased neurite length.	
			Decreased in serotonin receptor (5-HT 5B) and BNDF gene expression in brain cortex of F1 and F2 mice.	
	Pregnant C57Bl6/J mice exposed to NaAsO_2_ (100 mg/L) in drinking water starting 1 week before conception until birth.	Impaired spatial and episodic memory in adulthood. Impairments in fear conditioning performance.	Global hypo-acetylation at H3K9.	[171]
			Highly significant representation of KRAB transcription factors.	
			Decreased viability of astroglial cells.	
Perinatal exposure 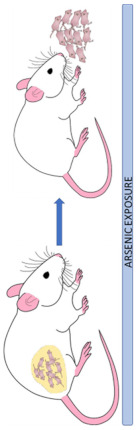	Pregnant rats—lactating dams—weaned pups exposed to NaAsO_2_ (100 mg/L) from GD 6 to PND 42	Alterations in learning and memory function.	Increased expression of glutamate decarboxylase and decrease the γ-aminobutyric acid transferase.	[172]
	Pregnant C57BL/6J mice—lactating dams and weaned pups exposed to 50 ppb of arsenic in drinking water from GD 1 to PND 23–28	Increased scape latencies. Increased immobility in a forced swim task. Induced spatial learning and memory impairment. Abnormal social behaviors, restricted interests, and repetitive behaviors (autism-like behaviors). Anxiety-like behaviors.	Reduced protein expression of CRFR_1._	[173,174,175,176]
			Increased sensitivity of dorsal hippocampal 5HT_1A_ receptor to serotonin.	
			Reduced corticosterone receptor in the hippocampus.	
			Decreased MAPK pathway transcripts, proteins and activity.	
			Decreased synaptic density in cortex, hippocampus, and cerebellum.	
			Decreased expression of markers for presynaptic and postsynaptic membranes (PSD-95 and SYP).	
	Pre-mating CD-1 mice–-Pregnant and lactating dams exposed to 20 mg/L of iAs: 1) from 30 days prior mating to postnatal day 15 and 2) from 30 days prior mating to PND 90.	Both iAs-PND15 and iAsPND90 decrease recognition of the object location indicating spatial memory impairment. Impaired learning and memory in the water maze test.	Increased expression of xCT and EAAC1 transporters on PND15.	[177,178]
			Down-regulation of NR2B NMDA receptor subunit on PND15.	
			Decreased GSH/GSSG ratio in cortex and hippocampus on PND 90.	
			Decreased GluA1 and GluA2 AMPA receptor subunits in hippocampus.	
			Low capacity to induce long-term potentiation and reduction in basal excitability.	
	Pregnant Kunming albino mice—lactating dams and weaned pups exposed to NaAsO_2_ (25–100 mg/L) in drinking water from GD 1 to PND 35.	Impaired spatial learning ability	Dose-dependent decreased on NR1, NR2A, NR2B, GluR1, GluR2, GluR3, CaMKII, and p-CaMKII expression in the hippocampus at PND 7, 14, 21 and 35.	[179,180]
			Decreased thickness of the postsynaptic density.	
			Decreased postsynaptic density protein-95 and synaptophysin proteins.	
	Pregnant Wistar rats—lactating dams and weaned pups exposed to NaAsO_2_ (0.05–0.10 mg/L) in drinking water from GD 0 to PND 35.	Impaired retention of long-term memory.	Decreased α7-nAChR expression.	[181]
			Decreased aspartate transaminase levels.	
			Increased glutamate levels.	
	Pregnant Sprague-Dawley rats—lactating dams—weaned pups exposed to NaAsO_2_ (23.6–71 ppm, drinking water) from GD 6 to weaning.	Delayed in bilateral eye opening and incisor eruption.	Decreased catalase activity.	[182]
		Poor slat board performance (PND 8–11).	No changes in brain dopamine and its metabolites.	
	Pregnant Sprague-Dawley rats—lactating dams—weaned pups exposed to NaAsO_2_ (2–4 mg/kg) from GD 6 to PND21	Hypoactivity.	Decreased mRNA and protein expression of dopamine D2 receptor.	[183]
		Impairment of spatial learning and memory	Altered dopamine and its metabolites in striatum.	
Early Postnatal exposure 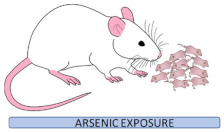	Wistar rat pups exposed to NaAsO_2_ (1.5–2 mg/kg, i.p.) from PND4 to PND17.	Impaired learning and memory	Decreased dendritic arborization and reduced number of spines in pyramidal neurons and granule cells.	[184]
Early life to adulthood 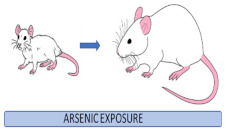	Weaning male rats exposed to NaAsO_2_ (68 mg/L) for 3 months.	Spatial memory damage.	Swollen mitochondrion and expanded rough endoplasmic reticulum in hippocampal neurons.	[185]
			Reduced mRNA expression of NMDA NR2A subunit.	
	Postnatal day 21 Wistar rats exposed to NaAsO_2_ (10 mg/kg, orally through gavage) for 1 week.	Decreased open-field behavior, locomotor activity, exploratory behavior, and grip strength.	Increased acetylcholine levels in cortex, cerebellum, and hippocampus.	[186]
Adult 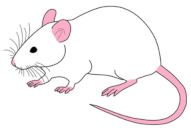	Male and female C57B1/6J mice exposed to NaAsO_2_ (0.05–50 mg As/L) in drinking water for 4 months.	Female mice showed hyperactivity each month after being exposed to 0.05, 0.5 and 5 mg As/L.	Dopamine decreased on the striatum and hypothalamus in female mice.	[187]
		Male mice showed hyperactivity in the group exposed to 0.5 mg As/L and hypoactivity in the group exposed to 50 mg As/L.	Tyrosine hydroxylase and cytosolic thioredoxin mRNA decrease in striatum and nucleus accumbens of male and female mice, respectively.	
	Adult male Swiss albino mice administered with As_2_O_3_ (2 mg/kg bw) through oral gavage for 45 days.	Enhanced anxiety levels.	Reduction in stratum pyramidale thickness (CA1).	[188]
		Reduced exploratory activity.		
			Decrease in density and size of pyramidal neurons.	
		Spatial memory retention impairment.		
			Reduction in hippocampal GSH levels	
		Fear for height and open environment.		
	Adult female Swiss albino mice administered with As_2_O_3_ (2 or 4 mg/kg bw) through oral gavage for 45 days.	Increased anxiety levels with both doses in open field test and elevates plus maze test.	Downregulation of estrogen receptor expression	[189]
		Reduced locomotion and decreased exploratory ability at both doses.		
			Reduction of BNDF and NMDAR 2B levels in hippocampus.	
		Impaired spatial learning and memory.		
	4-week-old male Kunming mice were administrated NaAsO_2_ (50 mg/kg bw) twice a week for 7 weeks.	Reduced spatial learning and memory ability.	Increased ROS levels and SOD activity in hippocampus.	[190]
			Loss of pyramidal cell layer.	
			Upregulated expression of SOD, HO-1 and GPx-3 genes.	
			Positive association between Nrf2/PPARγ expression in hippocampus.	
	9-weeks old male Kunming mice exposed to arsenic trioxide (4 ppm) in drinking water for 90 days.	Impairment of learning and memory.	Thickness postsynaptic density and synaptic cleft width decrease in hippocampus.	[191]
	9-weeks old male SPF mice exposed to As_2_O_3_ in drinking water for 60 days.	Poor performance in cognition functions.	Reduced thickness of cerebellar postsynaptic density.	[192,193]
			Decreased mRNA expressions of genes related to long-term potentiation and long-term depression.	
			Cellular edema in neurons with metamorphotic nuclei in hippocampus.	
			Reduction in cells body and number of pyramidal cells in CA3 area.	
	Male ICR mice exposed to realgar (90% As_2_S_2_; 0.15, 0.45 or 1.35 g/kg) once a day for 12 weeks.	Decreased locomotor activity and exploratory behavior.	Decreased SOD activity and increased MDA levels in brain cortex.	[194]
			Lost plasma membrane integrity. Swollen mitochondria, vague structure of rough endoplasmic reticulum and vacuoles structures presence in cortex neurons.	
			Decreased in the number of synaptic structures, in synaptic vesicles and in the curvature of the synaptic interface. Increase in the distance of synaptic cleft and in postsynaptic density.	
	Male C57/BL/6J exposed to As_2_O_3_ (10 mg/L) for 8 weeks.	Enhance anxiety-like behavior on elevated plus maze and open field test.	Decrease in BDNF-TrkB pathway.	[195]
		Increase depression-like behaviors on tail suspension test and forced swimming test in the reserpine pre-treated mice.		
	2-months old CD1 mice exposed to 0.5 or 5.0 mg As/L in drinking water for 6 months.	Hypoactivity.	Decrease in the expression of mRNA for dopamine receptors D2 in striatum.	[196]
		Alterations on rearing and on-wall rearing and barbering behaviors.		
	Swiss male Albino mice exposed to NaAsO_2_ (10 mg/kg body weight) in drinking water for 60 days.	Anxiety-like behaviors.	Increased blood indices of alkaline phosphatase, alanine aminotransferase, creatinine, aspartate aminotransferase, and urea.	[197,198]
		Deficits in spatial memory and learning.	Decreased butyryl cholinesterase and SOD activity.	
	Male C57BL/6J mice exposed to NaAsO_2_ (50 mg/L) for 24 weeks in drinking water.	Learning and memory impairments.	Activation of microglia in cortex and dentate gyrus in the hippocampus.	[199]
	Adult male Sprague Dawley rats exposed to NaAsO_2_ (5, 10, and 20 mg/Kg, intragastric via) for 2 or 2 weeks.	Reduced locomotor activity.	Alterations in monoamine content in midbrain and cortex.	[200]
		Increased numbers of errors in egocentric task.		
	Male Wistar rats exposed to NaAsO_2_ (1 mg/Kg, oral via) once daily for 6 months.	Loss of memory and learning ability.	Increased ROS levels, nitric oxide generation and lipid peroxidation levels.	[201]
			Decreased GSH levels.	
			Decreased GPx, GST, SOD and acetylcholinesterase activity in cortex and hippocampus.	
	Male Sprague-Dawley rats exposed to iAs (50 mg/L) in drinking water for a year.	Hypoactivity.	Increased striatal dopamine content. Up-regulation of mRNA for Mn-SOD and cytosolic thioredoxin enzymes. Down-regulation of dopamine receptor D2, D1 and Nrf2 mRNA expression.	[202]
	Adult male Wistar rats exposed to NaAsO_2_ (2mg/kg, orally through gavage) for 10 weeks.	Decreased exploratory behavior.	Increased lipoperoxidation in cortex, hippocampus, and cerebellum.	[203]
		Hypoactivity.		
			Decreased Mn-SOD, Cu/Zn-SOD, CAT, GPx, GR and GST activity in cortex, hippocampus and cerebellum.	
		Decreased grip strength of the fore limb and hind limb.		
	Adult male Wistar rats exposed to NaAsO_2_ (10 mg/kg, orally through gavage) for 6 weeks.	Decreased open field motility.	Increased latency of the cortical evoked potentials	[204]
			Decreased peripheral nerve conduction velocity.	
	Adult and aging Wistar rats exposed to NaAsO_2_ (10 mg/kg, orally through gavage) for 1 week.	Decreased the open-field behaviour (crossings, rearings, sniffings, and groomings), locomotor activity, grip strength, and exploratory behaviour.	Disturbances in cholinergic system.	[186]
		Increased escape latency of all the three phases observed in the water maze test.		
	Adult female Wistar rats exposed to NaAsO_2_ (13 mg/Kg, orally) for 3 weeks.	Suppressed exploratory behavior.	Induce oxidative stress.	[205]
		Decreased motor coordination.		
			Degenerative changes (vacuolization, inflammatory infiltration, karyosis, pyknosis, and necrosis) in the neuronal cells.	
		Decreased immobility period in the forced swim test.		
			Decreased dopamine, noradrenaline, and serotonin levels.	
		Decreased head dips count		
	Adult male Wistar rats exposed to As (10 mg/kg/day, in drinking water) for 21 days.	Impaired motor function, recognition learning and memory, spatial learning, and social interaction.	Decreased GSH and reduced antioxidant power.	[206]
		Increased anxiety-like behavior.	Increased lipid peroxidation.	

## Data Availability

Not applicable.
